# Selected reaction monitoring as an effective method for reliable quantification of disease-associated proteins in maple syrup urine disease

**DOI:** 10.1002/mgg3.88

**Published:** 2014-06-04

**Authors:** Paula Fernández-Guerra, Rune I D Birkler, Begoña Merinero, Magdalena Ugarte, Niels Gregersen, Pilar Rodríguez-Pombo, Peter Bross, Johan Palmfeldt

**Affiliations:** 1 Research Unit for Molecular Medicine, Department of Clinical Medicine, Aarhus University and Aarhus University HospitalAarhus, Denmark; 2 Centro de Diagnóstico de Enfermedades Moleculares (CEDEM), Centro de Investigación en Red de Enfermedades Raras (CIBERER), IDIPAZ, Universidad Autónoma MadridMadrid, Spain; 3 Dpto Biol. Mol., Centro de Diagnóstico de Enfermedades Moleculares (CEDEM), Centro de Biología Molecular Severo Ochoa, UAM-CSIC, Centro de Investigación en Red de Enfermedades Raras (CIBERER), IDIPAZ, Universidad Autónoma MadridMadrid, Spain

**Keywords:** BCKDH, branched-chain amino acid catabolism, liquid chromatography, maple syrup urine disease, mass spectrometry, MCAD (ACADM), MSUD, selected reaction monitoring, SRM, tandem mass spectrometry

## Abstract

Selected reaction monitoring (SRM) mass spectrometry can quantitatively measure proteins by specific targeting of peptide sequences, and allows the determination of multiple proteins in one single analysis. Here, we show the feasibility of simultaneous measurements of multiple proteins in mitochondria-enriched samples from cultured fibroblasts from healthy individuals and patients with mutations in branched-chain *α*-ketoacid dehydrogenase (BCKDH) complex. BCKDH is a mitochondrial multienzyme complex and its defective activity causes maple syrup urine disease (MSUD), a rare but severe inherited metabolic disorder. Four different genes encode the catalytic subunits of BCKDH: E1*α* (*BCKDHA*), E1*β* (*BCKDHB*), E2 (*DBT*), and E3 (*DLD*). All four proteins were successfully quantified in healthy individuals. However, the E1*α* and E1*β* proteins were not detected in patients carrying mutations in one of those genes, whereas mRNA levels were almost unaltered, indicating instability of E1*α* and E1*β* monomers. Using SRM we elucidated the protein effects of mutations generating premature termination codons or misfolded proteins. SRM is a complement to transcript level measurements and a valuable tool to shed light on molecular mechanisms and on effects of pharmacological therapies at protein level. SRM is particularly effective for inherited disorders caused by multiple proteins such as defects in multienzyme complexes.

## Introduction

Targeted proteomics using selected reaction monitoring (SRM) is a mass spectrometry (MS) -based methodology for precise and accurate quantification of proteins in complex mixtures. Quantitative measurement of proteins indicates changes in protein expression that help explain the functional states of enzymes, pathways, and networks (Gstaiger and Aebersold 2009). The “gold standard” methods are antibody-based methods such as ELISA (enzyme-linked immunosorbent assay) or Western blot analysis (Aebersold et al. 2013). However, these methods are often limited by the performance of the antibodies or the lack of availability of antibodies for certain proteins. In complex protein mixtures many antibodies unspecifically detect several targets and the correct target can then only be estimated from the molecular weight of the protein in Western blot analysis. Furthermore, some antibodies may not distinguish highly similar homologs or specific modifications (Liebler and Zimmerman 2013).

Targeted proteomics overcome many of the limitations of antibody-based methods and provide new capabilities for protein analysis (Aebersold et al. 2013; Liebler and Zimmerman 2013). The underlying concept is that proteins can be quantified by measuring their specific constituent peptides after proteolytic digestion. The acquisition of data only for specifically selected peptides allows measurements with high precision and high throughput. MS-based targeted protein assays offer two major advantages over immunoassays; first, the ability to systematically configure a specific assay for essentially any protein without the requirement of an antibody and second, the ability to perform multiplexed analysis of many peptides in a single analysis. This capability of multiplexed analysis allows systematic measurement of multiprotein complexes, networks, and pathways in one sample in a single analysis (Liebler and Zimmerman 2013). The identification of the targeted protein is achieved confidently due to the selection of peptides with sequences unique to the target protein, and the co-occurrence of multiple peptide fragment ions (transitions) that identify the targeted peptide sequence with high specificity. All these values are combined to assure with high probability that the targeted peptide has been detected. The peak areas for multiple transitions are integrated as measures of peptide abundance and serve as the basis for quantitative comparisons (Liebler and Zimmerman 2013).

To establish a correlation between genotype and phenotype is a challenge for any inherited disorder, but when known it provides important information for devising genotype-specific treatments (Weatherall 2001; Aebersold et al. 2013). Moreover, the majority of inherited metabolic disorders (IMDs) show moderate or very low correlation between genotype and phenotype (Rodriguez-Pombo et al. 2006; Gregersen et al. 2008; Olsen et al. 2013). IMDs are caused by defects in metabolic enzymes and some are enzymatic complexes formed by several protein subunits encoded by different genes, like branched-chain *α*-ketoacid dehydrogenase (BCKDH). This multiprotein nature adds another dimension to the genotype–phenotype association challenge. The defective activity of the BCKDH complex (EC 1.2.4.4.) causes maple syrup urine disease (MSUD) (MIM#248600), an autosomal recessive IMD of the catabolism of amino acids (Chuang et al. 2006). The BCKDH catalytic subunits are encoded by four different genes: E1*α* (*BCKDHA*) (MIM#608348), E1*β* (*BCKDHB*) (MIM#248611), E2 (*DBT*) (MIM#248610), and E3 (*DLD*) (MIM#238331) (Chuang et al. 2006). The E3 subunit is shared by the other mitochondrial ketoacid dehydrogenase complexes and the glycine cleavage system, therefore patients with E3 deficiency have a combined deficiency (MIM#246900) and are not considered as MSUD patients (Nellis and Danner 2000).

The etiology of MSUD is heterogeneous as different genes encode the BCKDH complex. Previous studies have shown the difficulty of establishing relationships between different mutations in the BCKDH complex and the clinical presentation of MSUD (Fisher et al. 1989; Nellis et al. 2003; Rodriguez-Pombo et al. 2006; Chuang et al. 2008). Moreover, pharmacological therapies have shown different responses in MSUD patients harboring different mutations in different genes, like the treatment with the osmolyte trimethylamine N-oxide (TMAO) or with phenylbutyrate (Song and Chuang 2001; Brunetti-Pierri et al. 2011). Furthermore, the protein subunits unique for the BCKDH are, in general, low-expressed in human dermal fibroblasts (HDFs), usually the only available cellular material from patients, which complicates their quantification by standard methods.

In this study, we show the measurement of the four protein subunits of the BCKDH complex by SRM in mitochondrial-enriched samples from cultured fibroblasts derived from healthy individuals and MSUD classic patients. We also studied a patient that harbors mutations in *ACADM* as well as *BCKDHA* gene leading to two different IMDs, medium-chain acyl-CoA dehydrogenase deficiency (MCADD) (MIM#201450) and MSUD, respectively. The mutation in the *ACADM* gene (c.985A>G) is a widely studied misfolding mutation that leads to the degradation of the protein medium-chain acyl-CoA dehydrogenase (MCAD) (EC 1.3.99.3) (MIM#607008) (Bross et al. 1994, 1995; Saijo et al. 1994). We exemplify how SRM can produce valuable data to fill the information gaps between gene variations, transcript levels, and enzyme activity. Moreover, this SRM assay can be used to analyze the effect of pharmacological therapies on the levels of BCKDH subunits, especially in the case of mutations leading to protein degradation or aggregation. Furthermore, the described methodological approach could also benefit other IMDs, especially those caused by defects in mitochondrial enzymes or multienzyme complexes.

## Materials and Methods

### Patients and healthy individuals

The patients studied are classic MSUD patients with BCKDH enzymatic activity below 3% with respect to the controls. Moreover, patient 4 has a combined defect of classic MSUD and MCADD. All four cases had a neonatal presentation, plasma leucine levels varied between 1800 and 3000 *μ*mol/L at diagnosis. In addition, patient 4 showed an increase in C8-carnitine and C8/C10 in neonatal dried blood spots by MS/MS. A summary of the patients' molecular data is shown in Table 1. The nomenclature of the mutations is according to the recommendations of Human Genome Variation Society (HGVS) (http://www.hgvs.org/mutnomen/) and has been revised with the software mutalyzer (https://mutalyzer.nl). The samples have been deidentified according to the regulations of the Danish Ethical Committee and the institutional Ethics Committee of the Universidad Autónoma de Madrid, ethical approval for the use of samples from patients was granted by the latter. HDFs from patients were obtained in accordance with the Helsinki Declaration of 1964, as revised in 2000. Fibroblasts from healthy individuals were used as controls and obtained from Camprex: cc2509 and from Coriell Cell Repositories (NJ, USA): GM08680, annotated NHDF-1 and NHDF-2, respectively.

**Table 1:** Genotype and phenotype information of the patients included in this study

Sample ID	Gene	Nucleotide change	Protein effect	Biochemical phenotype
Patient 1	*BCKDHA*	c. [117delC];[117dupC]	p. [Arg40Glyfs*23];[Arg40Glnfs*11]	Classic
Patient 2	*BCKDHB*	c. [853C>T];[853C>T]	p. [Arg285*];[Arg285*]	Classic
Patient 3	*BCKDHB*	c. [646A>G];[646A>G]	p. [Arg216Gly];[Arg216Gly]	Classic
Patient 4	*BCKDHA; ACADM*	c. [117delC];[117delC] c. [985A>G];[985A>G]	p. [Arg40Glyfs*23];[Arg40Glyfs*23] p. [Lys329Glu];[Lys329Glu]	Classic and MCADD

Gene bank reference sequences: NM_000709.3 for *BCKDHA*, NM_000056.2 for *BCKDHB*, and NM_000016.5 for *ACADM*.

### Cell culture

Cells were cultivated according to standard procedures. Briefly, cells were maintained in Dulbecco's modified Eagle's media (4.5 g/L of glucose) supplemented with 10% (v/v) fetal bovine serum, 2 mmol/L of l-glutamine, and 0.1% antibiotics (penicillin/streptomycin). Fibroblasts were cultivated until passage 13th in mycoplasma-free conditions and were collected at 80% confluence.

### RNA isolation and real-time PCR quantification

The mRNA expression levels for *BCKDHA* (NM_000709.3), *BCKDHB* (NM_000056.2), and *GAPDH* (NP_001276674) genes were assessed by performing reverse-transcription polymerase chain reaction quantification (qRT-PCR). The design of the RT-PCR primers was done using ProbeFinder software (Roche Applied Science, Indianapolis, IN), and probe selection with the Universal Probe Library (Roche Applied Sciences). Total mRNA isolation and qRT-PCR were performed as described previously (Alcaide et al. 2011). Relative quantification of gene expression was done by the comparative threshold cycle (Ct) method (Livak and Schmittgen 2001; Schmittgen and Livak 2008); ΔC_T_ was calculated as the C_T_ difference between the target gene and the housekeeping gene (*GAPDH*). The relative amount of transcripts in patients with respect to controls was calculated according to the formula: ΔΔC_T_ = ΔC_T_ (patient) − ΔC_T_ (control). The fold change for each gene was calculated as 2^−ΔΔCT^.

### Mitochondrial enrichment

At least 1.5 × 10^7^ cells were collected and resuspended in 10 mL 3-morpholinopropane-1-sulfonic acid (MOPS) buffer (10 mmol/L, pH 7.2) containing 200 mmol/L sucrose and 0.1 mmol/L Ethylenediaminetetraacetic acid (EDTA) with protease inhibitor cocktail tablets (Roche Diagnostic GmbH, Manheim, Germany). The cells were disrupted on ice by 30 strokes with a Dounce homogenizer followed by differential centrifugation as described before (Palmfeldt et al. 2009). The mitochondria-enriched pellets were resuspended in 0.5 mol/L triethylammonium bicarbonate buffer (pH 8.5) and treated with ultrasonication on ice water (Branson Sonifier 250, Branson ultrasonics corp, Danbury, USA) at output control 3 and 30% duty cycle for six rounds of five pulses with one on minute on ice between each round; followed by centrifugation at 10,000 × g for 10 min. Only soluble protein was used and concentrations were measured by the Bradford assay (Bio-Rad Laboratories, Hercules, CA).

### Sample preparation for SRM analysis

**Figure 1:**
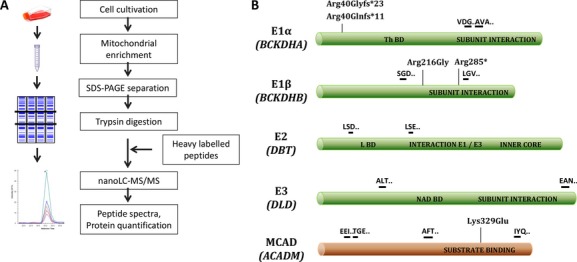
(A) Overall workflow of the process followed in the SRM assay. (B) Scheme of the proteins studied and the location of the selected peptides and mutations for each protein. The three letter code design the first three amino acids of each peptide, for E1*α*: VDG (VDGNDVFAVYNATK) and AVA (AVAENQPFLIEAMTYR); E1*β*: SGD (SGDLFNCGSLTIR) and LGV (LGVSCEVIDLR); E2: LSD (LSDIGEGIR) and LSE (LSEVVGSGK); E3: ALT (ALTGGIAHLFK) and EAN (EANLAASFGK); MCAD: EEI (EEIIPVAAEYDK), TGE (TGEYPVPLIR), AFT (AFTGFIVEADTPGIQIGR), and IYQ (IYQIYEGTSQIQR). Th BD: thiamine-binding domain; L BD: lipoyl-binding domain; NAD BD: NAD-binding domain.

The workflow followed is outlined in Figure 1A. Protein extract from mitochondria-enriched samples from patients and healthy individuals (80 *μ*g from each) were separated on polyacrylamide gel electrophoresis (SDS-PAGE) (Criterion™ XTG Any kD™, Bio-Rad). From each lane, the central area (25–60 kDa) that includes the proteins of interest (E1*α*, 50 kDa; E1*β*, 43 kDa; E2, 53 kDa; E3, 54 kDa; MCAD, 47 kDa) was cut and processed by in-gel digestion protocol as previously described (Hansen et al. 2011).

### Selection of targeted peptides

Peptide selection, method development, and data analysis were performed using the software Skyline (MacLean et al. 2010). Detail description and the list of peptides used can be found in the Data S1. Briefly, two to four peptide candidates for each protein were selected based on uniqueness in the whole human proteome (Uniprot database version 2012_06_19 containing 20,202 reviewed sequences) (Magrane and Consortium 2011) and high-intensity y-fragment ions in the spectral libraries, in-house, or NIST database (National Institute of Standards and Technology, http://peptide.nist.gov/). Finally, the five most intense transitions were selected for each peptide. The synthetic tryptic peptides (SpikeTide™ from JPT Peptide Technologies, Berlin, Germany) were heavily labeled at the amino acid residues lysine (+8 Da) or arginine (+10 Da) and cysteines were carbamidomethylated. A stock solution of heavy-labeled peptide standards at 167 nmol/L was prepared in buffer A (H_2_O, 2% AcN, 0.1% HCOOH). A working solution at 20 nmol/L was used.

### Analysis on triple quadrupole instrument

The liquid chromatography (LC)-MS system consisted of a Proxeon EASY nano-LC (Proxeon, Odense, Denmark) coupled to a TSQ-Vantage triple quadrupole (QQQ) mass spectrometer (Thermo Fisher Scientific, Waltham, MA). The LC was C18-based reverse phase separation with a 2-cm trap column and a 10-cm analytical column (EASY column, Thermo). Columns were equilibrated with 100% buffer A and peptides were eluted with a 40 min linear gradient of buffer A and buffer B (AcN, 5% H_2_O, 0.1% HCOOH), until 40% B was reached. MS raw data files were imported into Skyline and each file was quality checked by visual inspection of retention time and ranking of transitions for each peptide. A more detail description can be found in the Data S1.

### SRM data normalization

The summed fragment ion peak areas were normalized to the corresponding signal responses from a spiked heavy-labeled peptide standard (see Data S1 for detailed explanation). This ratio to standard for each peptide was subsequently normalized to a mitochondrial reference protein (Hsp60) to correct for the amount of mitochondrial protein in each sample. This ratio to Hsp60 for each protein from the patient's samples was then compared to the corresponding ratio in the samples from healthy individuals.

### Detection level

The detection level for the E1*α*, E1*β*, and MCAD proteins was defined as the background level for each protein. The background levels, from analytical noise, for these proteins were determined in the samples from healthy individuals (*n* = 12) using one peptide for each protein: VDGNDVFAVYNATK (E1*α*, *BCKDHA*), LGVSCEVIDLR (E1*β, BCKDHB*), and EEIIPVAAEYDK (MCAD, *ACADM*). These peptides were selected according to strict criteria (see Selection of targeted peptides) to obtain robust quantification. The peaks were integrated in Skyline and the peak areas as well as background values were exported to Excel. The 95% interval of the background noise for each peptide was calculated as the sum of 1.645 times the standard deviation (*n* = 12) of the background values and the average background value (Armbruster and Pry 2008).

## Results

SRM MS is a strong tool to quantify disease-associated proteins. The high sensitivity and analytical precision of SRM allows the detection of proteins at low concentrations in complex mixtures (Calvo et al. 2011; Picotti et al. 2013). In this study, we applied SRM to quantify the four subunits of BCKDH and an additional protein (MCAD) in HDFs from healthy individuals as controls and patients with MSUD. The four selected patients show the classic phenotype that accounts for 70% of the MSUD patients and is associated with high mortality if not treated early (Chuang et al. 2006). Moreover, patient 4 also shows MCADD caused by the prevalent mutation (p.Lys329Glu) leading to protein misfolding and instability (Bross et al. 1994, 1995; Saijo et al. 1994).

### Analysis of samples from MSUD patients and healthy individuals

**Figure 2:**
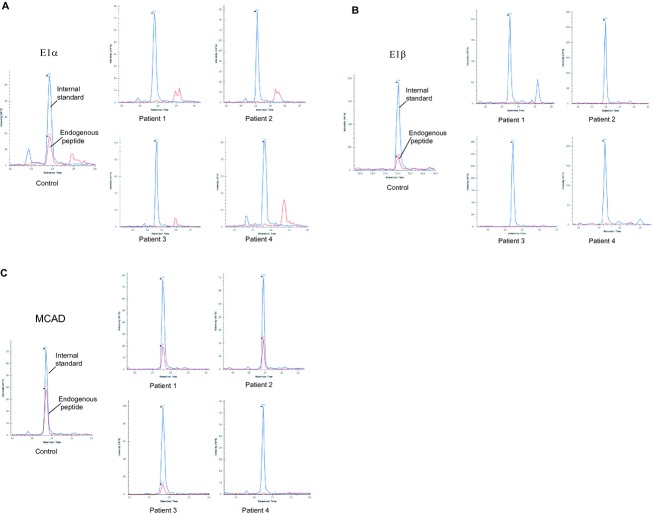
Analysis of samples by SRM. Extracted chromatograms derived from the injection of peptide extracts are shown, where the blue color corresponds to the internal standard (heavy-labeled peptides) and the red color to the endogenous peptide. For quantification purposes the ratio endogenous peptide to internal standard was used. The *y*-axis corresponds to the intensity and the *x*-axis to the retention time. (A) Representative peptide analysis corresponding to the E1*α* (*BCKDHA*) subunit of the BCKDH complex. The peptide VDGNDVFAVYNATK was detected and quantified in the samples from healthy individuals (chromatogram on the left), but not in the samples from patients (chromatograms on the right). The detection level for the E1*α* subunit was determined as 23% respect to controls, so the E1*α* subunit levels in the samples from patients are below 23%. (B) Representative peptide analysis corresponding to the E1*β* (*BCKDHB*) subunit of the BCKDH complex. The peptide LGVSCEVIDLR was detected and quantified in the samples from healthy individuals (chromatogram on the left), but not in the samples from patients (chromatograms on the right). The detection level for the E1*β* subunit was determined as 19% respect to controls. Therefore, the E1*β* subunit levels in the samples from patients are below 19%. (C) Representative peptide analysis corresponding to the MCAD (*ACADM*) protein. The peptide IYQIYEGTSQIQR was detected and quantified in the samples from healthy individuals (chromatogram on the left) and in patients 1, 2, and 3, but not in the samples from patient 4. The detection level for the MCAD protein was determined as 9% respect to controls. Thus, the MCAD protein levels in the sample from patient 4 are below 9%. Two different healthy individuals were analyzed with three biological replicates for each of them. The detection levels for each protein were determined from the background noise in all control samples (*n* = 12).

We applied the procedure described in Figure 1A on the HDF samples. The positions of selected peptides in the proteins are shown in Figure 1B. In all experiments, SRM transitions from endogenous peptides were confirmed by chromatographic co-elution with heavy-labeled peptides (Fig. S1). One of the advantages of SRM-based assays is that multiple proteins can be analyzed in a single analysis. In our method, five proteins associated with IMDs were analyzed together with a mitochondrial reference protein, Hsp60, as a “loading control.” The Hsp60 levels were shown to be stable, that is, not being influenced by the MSUD pathogenicity in HDFs from patients (Fig. S2). The results of the SRM assay of four samples from MSUD patients and two healthy individuals are shown in Table 2. In all samples the reference protein Hsp60, the E3 subunit, and the E2 subunit were detected and quantified. However, E1*α* and E1*β* subunits were detected only in the samples from healthy individuals, meaning that both subunits were decreased below detection levels in all patient samples even though only one of the encoding genes carried a disease-causing gene variation. Chromatograms of selected peptides for the subunit E1*α* and E1*β* are shown in Figure 2A and B, respectively. The presence of the endogenous peptide (red) can only be observed in the sample from healthy individuals and not in any of the samples from patients. The presence of the heavy-labeled peptide peak (blue) confirms that the SRM assay for that peptide was successful. The detection level, defined as 95% of the background signal (see Materials and Methods), was calculated to be below 23% and 19% of the levels detected in the healthy individuals for the E1*α* and E1*β* subunit, respectively. These results indicate that the levels of the E1*α* and E1*β* subunit were below 23% and 19%, respectively, in all samples from the four MSUD patients studied.

**Table 2:** Protein levels in human dermal fibroblasts from classic MSUD patients normalized by Hsp60 and represented as percentage respect to the healthy individuals

	Protein levels normalized by Hsp60				
		
Sample	E1*α*	E1*β*	E2 (%)	E3 (%)	MCAD (%)
Patient 1	n.d	n.d	152	110	181
Patient 2	n.d	n.d	102	106	89
Patient 3	n.d	n.d	136	124	124
Patient 4	n.d	n.d	100	110	n.d

n.d., not detected, denotes measurements that were below 95% confidence range of background noise, which was calculated to be 23%, 19%, and 9% of protein level in samples from healthy individuals for E1*α* (*BCKDHA*), E1*β* (*BCKDHB*), and MCAD (*ACADM*) respectively.

Patients 1 and 2, with mutations in the *BCKDHA* and *BCKDHB* genes, respectively, harbor nucleotide changes that generate premature stop codons (PTCs). PTCs can activate the nonsense-mediated decay (NMD) pathway that eliminates transcripts with PTC. We measured the relative RNA abundance of the *BCKDHA* and *BCKDHB* messengers in HDFs from these patients by RT-PCR. Patient 1 harbors PTCs (p.[Arg40Glyfs*23 + Arg40Glnfs*11]) in the E1*α* subunit that could activate NMD and induce the degradation of the *BCKDHA* mRNA. However, the relative amount of *BCKDHA* mRNA was only decreased by approximately 26% with respect to the controls, and the *BCKDHB* mRNA amount was comparable to the healthy individuals (Fig. S2). Similarly, there was no apparent sign of NMD for *BCKDHB* in HDFs from patient 2 that harbors a PTC (p.Arg285*) in the E1*β* subunit. The relative amount of *BCKDHB* mRNA was only decreased approximately 14% and the *BCKDHA* mRNA amount was similar as that in controls. These results showed that the absence of protein for the E1*α* and E1*β* subunits in these patients is caused by events at the posttranscriptional level.

Patient 3 harbors a missense mutation (c.646A>G) in the *BCKDHB* gene that could compromise the stability of the E1*β* subunit. As it is shown in Figure 2B the E1*β* endogenous peptide was indeed missing while the heavy-labeled peptide was detected. This indicates that the E1*β* subunit level was below 19% of the level from the controls. As observed in patients 1 and 2, the E1*α* subunit was below 23% of the value in the controls, indicating instability of both monomers of E1.

In the case of the MCAD protein, it was detected in all samples except for the sample from patient 4 (Fig. 2C). Patient 4 harbors two different gene variations resulting in both MSUD and MCADD. Patient 4 harbors a PTC mutation (c.117delC) in both alleles of the *BCKDHA* gene (the same as patient 1) and a missense mutation (c.985A>G) in both alleles of the *ACADM* gene. The mutant MCAD protein is either absent or at least below detection level, which was estimated to be 9% of the levels measured in controls (Fig. 2C). The E1*α* and E1*β* were not detected in samples from patient 4 in the SRM assay (Fig. 2A and B). This corroborates the results obtained with patient 1 (with the same E1*α* mutation) and shows the reproducibility of the SRM assay in different biological backgrounds. Moreover, the absence of MCAD protein in patient 4 is in concordance with previous studies of this particular mutation in the *ACADM* gene (Coates et al. 1992; Bross et al. 1994).

## Discussion

Accurate quantification of proteins in complex mixtures can be done by targeted proteomics using MS and SRM MS is highly suitable for this task (Calvo et al. 2011). The high sensitivity and analytical precision of SRM allows the detection of proteins at low concentrations and can be applied to quantify disease-associated proteins (Picotti et al. 2013). Despite the high selectivity and specificity of the SRM, peptides and other small molecules in the biological matrix can interfere in the mass-to-charge (m/z) channels monitored, resulting in inaccurate quantitative measurements (Duncan et al. 2009; Sherman et al. 2009). The main limitation of targeted approaches is the extremely large concentration range and complexity of the proteins in the matrix analyzed. Among the different methods for enrichment of proteins we choose mitochondrial enrichment because it is a standard method for mitochondrial protein studies including MS approaches, and it removes the most abundant cytosolic proteins that could compromise the detection of the lower expressed mitochondrial proteins (Palmfeldt et al. 2009; Gregersen et al. 2012). We added a second step to reduce the complexity of the sample, gel fractionation. The samples were run in an SDS-PAGE that was cut in pieces and the piece from 25 to 60 kDa was used for the analysis. This fractionation leads to decreased sample complexity and reduction in the background noise, favoring the SRM assay.

### Evaluation of SRM assay design

Heavy-labeled synthetic peptides can serve as internal standard for targeted proteomics, increasing the confidence of peptide identification and facilitating relative quantification (Picotti and Aebersold 2011; Liebler and Zimmerman 2013). The signal from the heavy-labeled synthetic peptides is used to normalize the different detection levels obtained by different endogenous peptides. The heavy-labeled synthetic peptides were selected according to strict criteria in order to avoid false positives (see Materials and Methods; and for positions of peptides in the protein sequence, see Fig. 1B). The heavy-labeled peptides were pooled in equimolar concentrations to obtain a standard mixture of heavy peptides that subsequently could be spiked into the analytical samples. Initially this standard mixture was analyzed by SRM to validate that the heavy peptide mixtures were lacking contamination from unlabeled peptides. Thereafter, a “training sample set” of mitochondria-enriched samples from control HDFs was used to evaluate the SRM assay design. The reference protein Hsp60, MCAD, and the four BCKDH subunits were successfully detected and the best transitions and peptides were chosen for further quantification of the samples.

### Analysis of samples from MSUD patients and healthy individuals

Four MSUD patients with classic phenotype, which accounts for 70% of the MSUD patients, were selected. These patients carry different mutations in the *BCKDHA* and *BCKDHB* genes that have been described previously (Rodriguez-Pombo et al. 2006). The mutations comprise early and late PTCs and missense changes. Whether PTCs mutations trigger NMD or lead to synthesis of proteins is not always easy to predict, therefore study of these mutations at mRNA and protein level can add information that could explain the phenotype of the patients. Moreover, the effect of missense mutations can be predicted with bioinformatics tools, however, these data should be used cautiously and verified by experimental approaches like the one showed in this study. In all samples the reference protein Hsp60, the E3 subunit, and the E2 subunit were detected and quantified. However, E1*α* and E1*β* subunits were detected only in the samples from healthy individuals (Fig. 2). These results indicate that the levels of the E1*α* and E1*β* subunit were below the detection levels of 23% and 19%, respectively, in all samples from the MSUD patients studied. Below that level, the SRM assay has not enough confidence to identify and quantify those peptides. The presence of the heavy-labeled peptide peak (blue) confirms that the SRM assay for that peptide was successful.

Patients 1 and 2 harbor nucleotide changes that generate PTCs. PTCs can activate the NMD pathway; a phylogenetically, widely conserved mechanism to eliminate transcripts with PTC and suppresses accumulation of C-terminal truncated peptides. However, sometimes mRNAs harboring PTCs escape NMD (Maquat 2004). Since the transcripts levels of *BCKDHA* and *BCKDHB* were not decreased, that is, not affected by NMD, the absence of protein for the E1*α* and E1*β* subunits in these patients is caused by events at the posttranscriptional level. On the other hand, patient 3 harbors a missense mutation (c.646A>G) in the *BCKDHB* gene and previous structural studies show the location of this residue in the inner core of the E1*β* subunit (Rodriguez-Pombo et al. 2006). This amino acid change could compromise the stability of the region triggering proteolytic degradation, which is in line with the fact that E1*β* was not detected in the present study. The lack of E1*β* resulted in protein levels below the detection level of the partner protein E1*α*, supporting the hypothesis that the two subunits of E1 are only stable when they are in complex with each other.

Previous studies have also suggested that mutations in either E1*α* or E1*β* influence the levels of the other subunit since these two proteins must assume their tetrameric structure to stabilize each other against degradation (Fisher et al. 1989, 1991; Chuang et al. 2008). This instability has also been studied in the crystal structure of the BCKDH complex where it is shown that E1*α* and E1*β* have large hydrophobic areas in the interacting regions between E1*α* and E1*β* (Aevarsson et al. 1999). The instability associated to E1*α* and E1*β* as monomers could explain why these proteins need the assistance of GroEL, in vitro, for folding each monomer and the heterodimers (Song et al. 2000; Wynn et al. 2000; Chuang et al. 2008). Moreover, treatment with “chemical chaperones” like the TMAO is capable of rescuing assembly defects at the step of the trapped heterodimeric ensemble (Song and Chuang 2001). All together, these findings are consistent with our results using the SRM assay. Moreover, it is well established that missense mutations in many proteins cause misfolding and lead to protein degradation or aggregation, and in either case the protein would be absent in the soluble fraction (Gregersen et al. 2006).

Patient 4 harbors a PTC mutation (c.117delC) in both alleles of the *BCKDHA* gene (same as allele 1 in patient 1) and a missense mutation (c.985A>G) in both alleles of the *ACADM* gene. The latter mutation has a high frequency among the MCADD patients and it is well studied to affect protein folding (Bross et al. 1994, 1995; Saijo et al. 1994). Our results obtained with the SRM assay corroborate these previous studies, since the unstable mutant protein here escaped detection (i.e., <9% of level in healthy individuals). Moreover, these results on MCAD protein exemplify the capability of the SRM assay to incorporate more proteins to a previously designed method.

Our results show that the lack of activity of the BCKDH complex in these patients, below 3% in all cases, is due to the absence of the catalytic subunits E1*α* and E1*β* besides unaltered transcripts levels. The monomers of E1*α* and E1*β* form an *αβ* heterodimer, that is further associated with another *αβ* heterodimer, that forms the active E1 subunit, that catalyses the decarboxylation of the branched-chain *α*-ketoacids. This is the first step of the reaction and the entrance of the substrate, without it the catabolism of branched-chain amino acids cannot continue further down in the metabolic pathway.

One potential limitation of this SRM assay is the sample material required for the assay. It is necessary to start with approximately 1.5 × 10^7^ cells, which in the case of HDFs takes weeks to collect and can be difficult or even impossible for HDFs derived from some patients. This high amount of cells is required for an efficient enrichment of mitochondria and for reaching sufficient quantities of mitochondrial proteins. The use of enrichment methods is valuable since they not only increase the concentration of our peptides of interest but also reduce the level of potentially interfering peptides.

By using this SRM assay we successfully detected and quantified low-expressed mitochondrial proteins that are involved in the pathogenesis of MSUD and MCADD. This method does not require antibodies that sometimes lead to unspecific or ambiguous results. The described SRM protein analysis can shed light on molecular mechanisms as well as on pharmacological therapies that influence the levels of key proteins in MSUD and MCADD. Furthermore, other extended SRM assays including additional proteins of interest such as other enzymes of amino acid metabolic pathways or marker proteins of stress in MSUD would be relatively straightforward and inexpensive to develop. The heavy-labeled peptides required as internal standards are readily available, whereas generating antibodies is a more costly and difficult process. Moreover, the levels of specific mutations in proteins (including point mutations) can be quantified using specific heavy-labeled peptides for each mutation, whereas antibodies for specific mutation are usually not possible to obtain. SRM methodology is also an important complement to the measurement of transcript level and a valuable tool to determine whether a certain gene variation causes a lack of protein or gain of function effects. Furthermore, this method could be applied to other IMDs and it is especially suited for those caused by defects in multienzyme complexes.

## Supporting Information

**Figure S1.** Analysis of SRM transitions for peptide identification. Extracted chromatograms derived from the injection of the protein extract are shown, where the blue color corresponds to the internal standard (heavy-labeled peptide) and the red color to the endogenous peptide. The *y*-axis corresponds to the intensity and the *x*-axis to the retention time. The transitions between the heavy-labeled peptide and the endogenous peptide show similar ranking especially in the most intense transitions. (A) Chromatograms of the peptide VDGNDVFAVYNATK for the identification of E1*α* (*BCKDHA*). (B) Chromatograms of the peptide LGVSCEVIDLR for the identification of E1*β* (*BCKDHB*). (C) Chromatograms of the peptide LSDIGEGIR for the identification of E2 (*DBT*). (D) Chromatograms of the peptide ALTGGIAHLFK for the identification of E3 (*DLD*). (E) Chromatograms of the peptide IYQIYEGTSQIQR for the identification of MCAD (*ACADM*). (F) Chromatograms of the peptide GYISPYFINTSK for the identification of Hsp60 (*HSPD1*). **Figure S2.** Analysis of the relative levels of the *BCKDHA* and *BCKDHB* transcripts in the patients 1 and 2. Transcript levels of HDFs from healthy individuals (*n* = 12) and the two patients (*n* = 3 each) were studied and the average of these levels were used to determine the ratio patients versus controls (C vs. P). **Data S1.** Supplementary Methods
