# Supplementary material for: Selected reaction monitoring as an effective method for reliable quantification of disease-associated proteins in maple syrup urine disease
Source: Mol Genet Genomic Med. 2014 Jun 4;2(5):383–92. doi: 10.1002/mgg3.88 (PMC4190873; doi:10.1002/mgg3.88)
Supplement: Supplementary file 1 [file mgg30002-0383-SD1.docx]

**
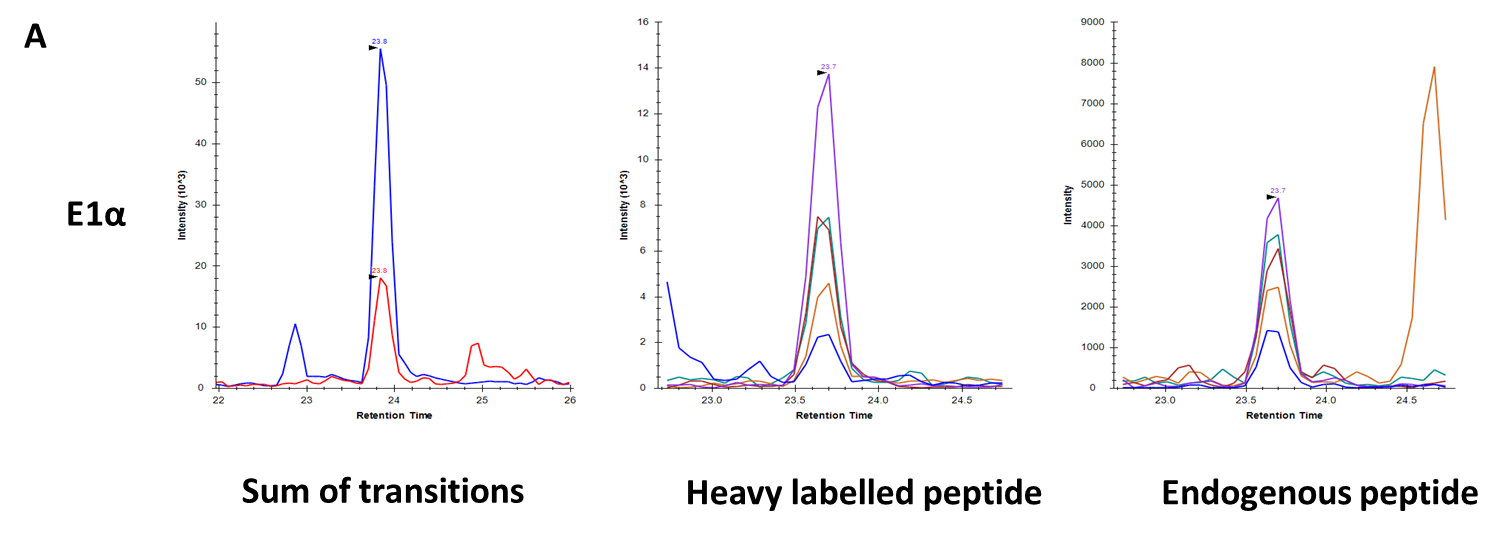
Supplementary Figure S1**

**
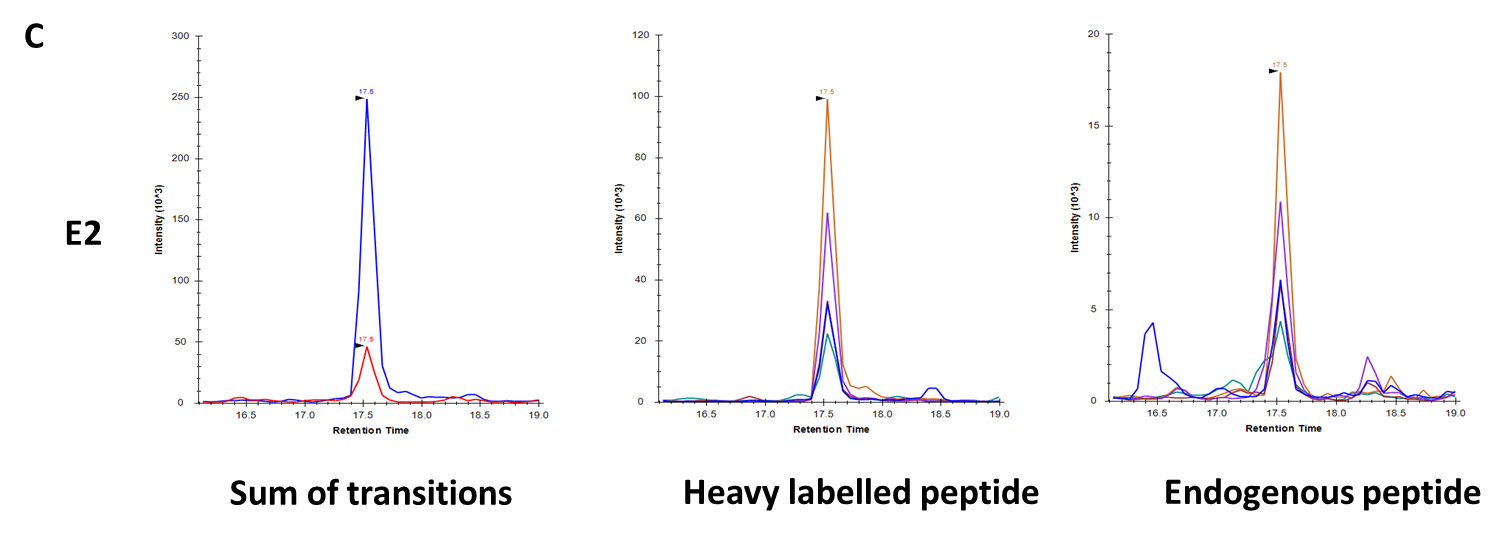

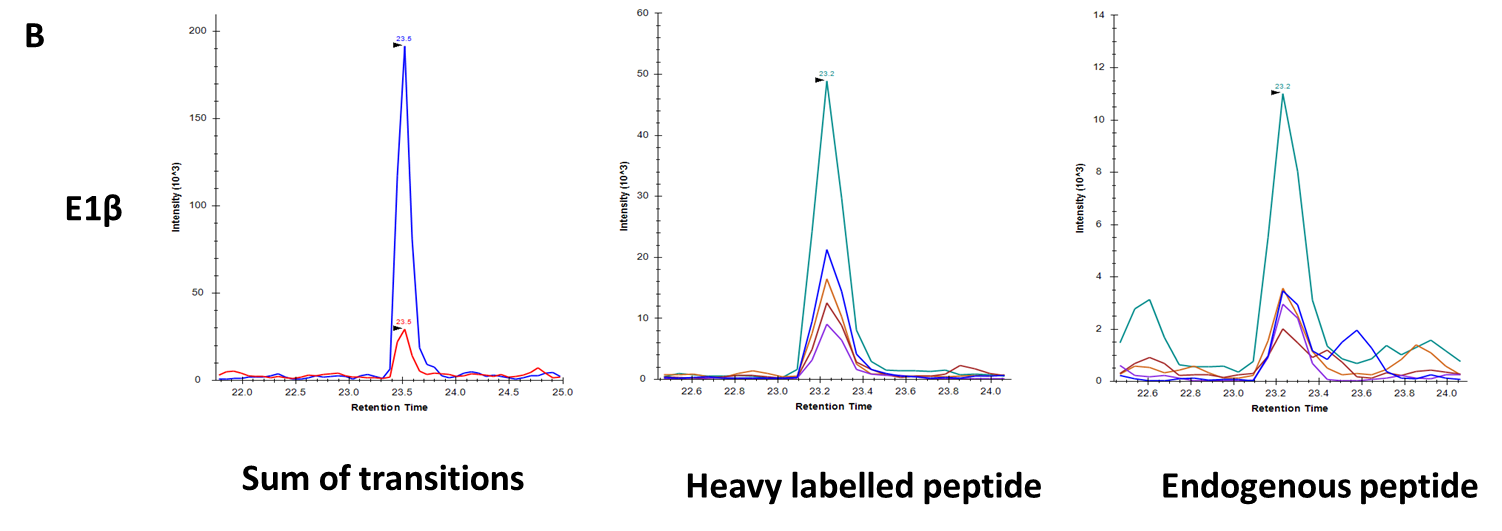
**

**
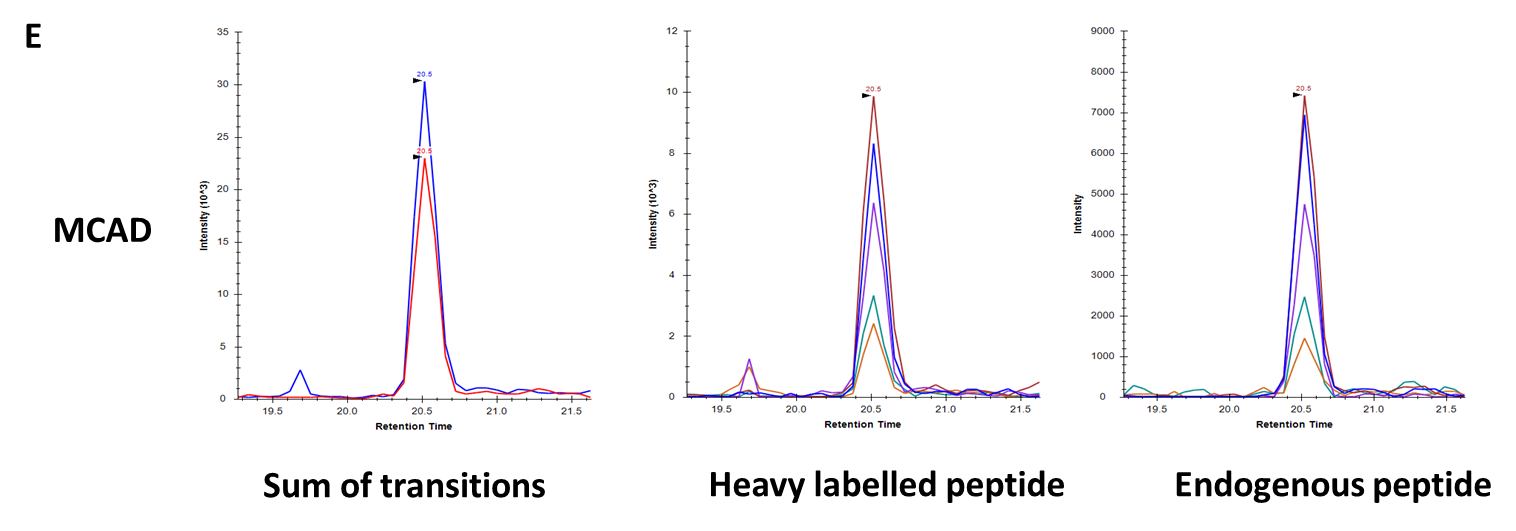

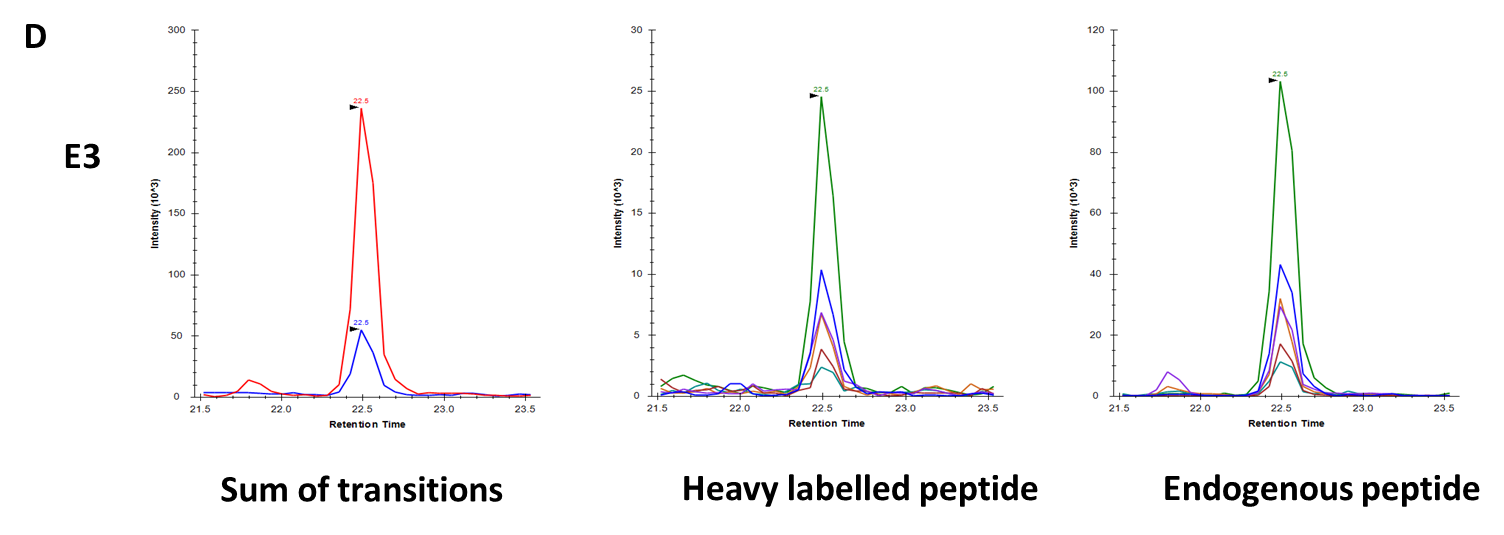
**

**
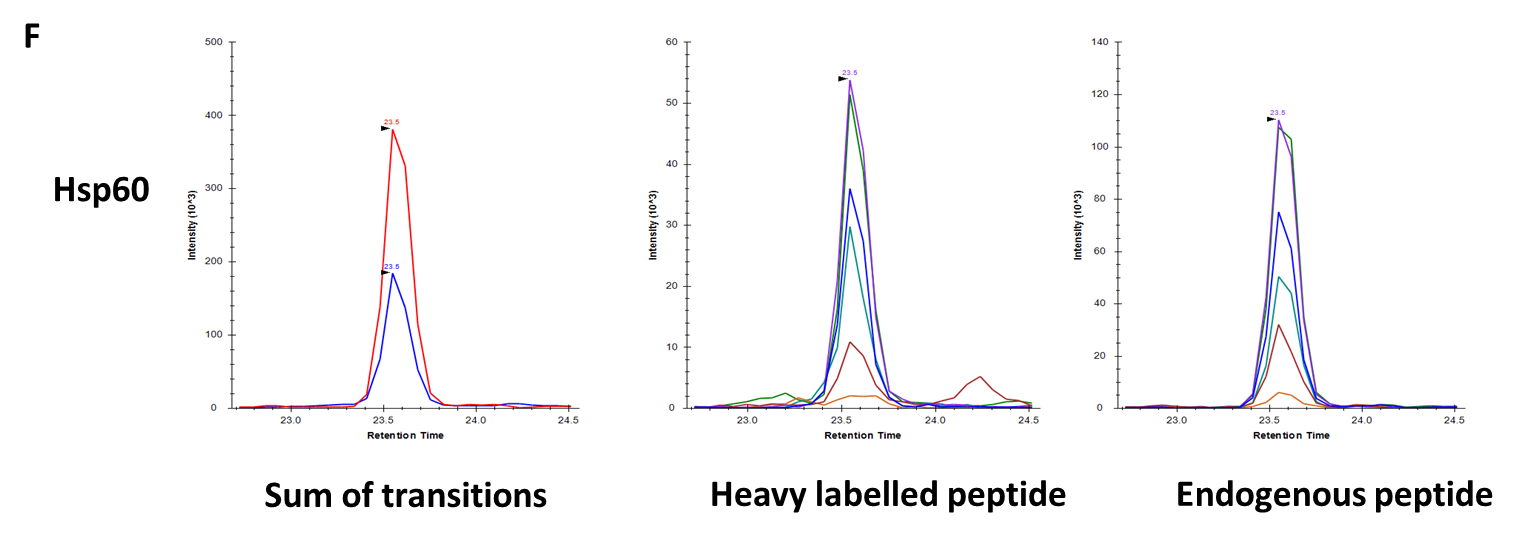
**

**Supplementary Figure S1.** Analysis of SRM transitions for peptide identification. Extracted chromatograms derived from the injection of the protein extract are shown, where the blue colour corresponds to the internal standard (heavy-labelled peptide) and the red colour to the endogenous peptide. The y-axis corresponds to the intensity and the x-axis to the retention time. The transitions between the heavy labelled peptide and the endogenous peptide show similar ranking especially in the most intense transitions. **(A)** Chromatograms of the peptide VDGNDVFAVYNATK for the identification of E1α (*BCKDHA*). **(B)** Chromatograms of the peptide LGVSCEVIDLR for the identification of E1β (*BCKDHB*). **(C)** Chromatograms of the peptide LSDIGEGIR for the identification of E2 (*DBT*). **(D)** Chromatograms of the peptide ALTGGIAHLFK for the identification of E3 (*DLD*). **(E)** Chromatograms of the peptide IYQIYEGTSQIQR for the identification of MCAD (*ACADM*). **(F)** Chromatograms of the peptide GYISPYFINTSK for the identification of Hsp60 (*HSPD1*).

**Supplementary Figure S2**

**
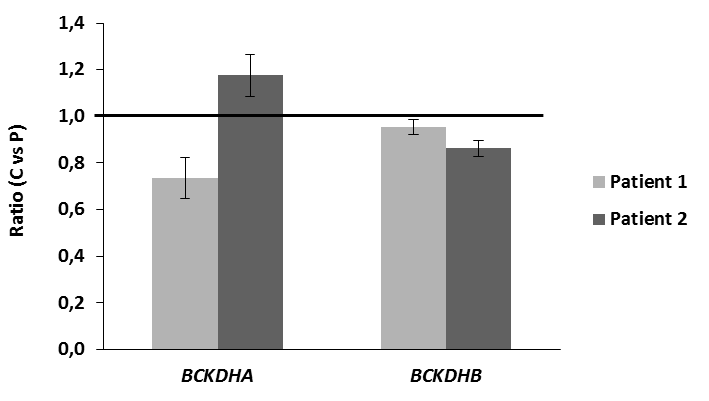
**

**Supplementary Figure S2.**  Analysis of the relative levels of the *BCKDHA* and *BCKDHB* transcripts in the patient 1 and patient 2. Transcript levels of HDFs from healthy individuals (n = 12) and the two patients (n = 3 each) were studied and the average of these levels were used to determine the ratio patients versus controls (C vs P)

**Supplementary Methods**

*Selection of targeted peptides*

Peptide selection, assay development, and data analysis were performed using the software Skyline (MacLean et al. 2010). Proteins selected for SRM validation were imported to Skyline and tryptic *in silico* digestions were carried out. From each protein two to four peptide candidates were further selected based on a series of selection criteria. The first requirement was uniqueness in the whole human proteome (Uniprot database version 2012_06_19 containing 20,202 reviewed sequences) (Magrane and Consortium 2011). Second, presence in spectral library databases generated from in-house data from previous studies or from the publicly available NIST database (National Institute of Standards and Technology, http://peptide.nist.gov/) with high intensity fragments ions of the y-series product ions. Third, using a built-in filter feature in the Skyline software, the five most intense transitions were selected for each peptide. Synthetic, crude, isotopically labelled peptide analogues (SpikeTide™) for each of the selected peptides were purchased from JPT Peptide Technologies, Germany. The tryptic peptides were heavily labelled at the amino acid residues Lysine (+8 Da) or Arginine (+10 Da). Peptides with cysteine residues were purchased as carbamidomethylated cysteines. Furthermore, during method development and optimization the numbers of protein specific peptides were adjusted to the peptides that showed good chromatography as well as sensitivity on the liquid chromatography-MS (LC-MS). The six proteins of interest for this study are listed below together with the targeted peptides passing the described quality criteria. In addition, peptides from six other proteins: **HSPA9,** ETFA, VDAC1, ETFB, SOD2 and CLPP, were also assayed to test the capability of the method, but the results are not described in this manuscript. Retention times for all peptides were determined and used to prepare the scheduled SRM assay with SRM time-windows of 8 minutes.

**The following specific peptides of the six proteins of interest were included in the SRM assay:**

**sp|P12694|ODBA_HUMAN** *2-oxoisovalerate dehydrogenase subunit alpha, mitochondrial OS=Homo sapiens GN=BCKDHA PE=1 SV=2*,

VDGNDVFAVYNAT**K** (charge state++, product ions y9+, y8+, y7+, y6+, y5+)

AVAENQPFLIEAMTY**R** (charge state++, product ions y10+, y7+, y6+, y5+, y3+, y14++)

**sp|P21953|ODBB_HUMAN** *2-oxoisovalerate dehydrogenase subunit beta, mitochondrial OS=Homo sapiens GN=BCKDHB PE=1 SV=2*,

SGDLFN**C**GSLTI**R** (charge state++, product ions y9+, y8+, y7+, y6+, y3+)

LGVS**C**EVIDL**R** (charge state++, product ions y8+, y7+, y4+, y3+, y2+)

**sp|P11182|ODB2_HUMAN** *Lipoamide acyltransferase component of branched-chain alpha-keto acid dehydrogenase complex, mitochondrial OS=Homo sapiens GN=DBT PE=1 SV=3*,

LSDIGEGI**R** (charge state++, product ions y8+, y7+, y6+, y5+, y3+)

LSEVVGSG**K** (charge state++, product ions y8+, y7+, y6+, y5+, y4+)

**sp|P09622|DLDH_HUMAN** *Dihydrolipoyl dehydrogenase, mitochondrial OS=Homo sapiens GN=DLD PE=1 SV=2*,

ALTGGIAHLF**K** (charge state++, product ions y9+, y8+, y7+, y5+, y4+, y9++)

EANLAASFG**K** (charge state++, product ions y8+, y6+, y5+, y4+, y3+)

**sp|P11310|ACADM_HUMAN** *Medium-chain specific acyl-CoA dehydrogenase, mitochondrial OS=Homo sapiens GN=ACADM PE=1 SV=1*,

EEIIPVAAEYD**K** (charge state++, product ions y10+, y9+, y8+, y5+, y8++)

TGEYPVPLI**R** (charge state++, product ions y7+, y6+, y4+, y2+, y7++)

AFTGFIVEADTPGIQIG**R** (charge state++, product ions y11+, y10+, y8+, y7+, y4+)

IYQIYEGTSQIQ**R** (charge state++, product ions y9+, y8+, y7+, y4+, y3+)

**sp|P10809|CH60_HUMAN** *60 kDa heat shock protein, mitochondrial OS=Homo sapiens GN=HSPD1 PE=1 SV=2*,

GYISPYFINTS**K** (charge state++, product ions y9+, y8+, y4+, y10++, y9++, y8++)

NAGVEGSLIVE**K** (charge state++, product ions y10+, y9+, y8+, y7+ y4+, y3+)

*Analysis on Triple Quadrupole Instrument*

Lyophilized samples were dissolved in 35 µL Buffer A (H_2_O, 2% AcN, 0.1% HCOOH). 1 µL of each sample was pooled and further mixed with heavy labelled peptides to a final concentration of 3.2 nM. This sample mixture was injected to check retention time, chromatography, and presence of the peptides. Following assay optimization, the samples were analysed in two separate series. The individual samples were mixed with 2.5 µL of the heavy-labelled peptides working solution and then, 10 µL was injected.

The LC-MS system consisted of a Proxeon EASY nano-LC (Proxeon, Odense, Denmark) coupled to a TSQ-Vantage triple quadrupole mass spectrometer (Thermo Fisher Scientific, Waltham, MA, USA). The LC was operated as C18 based reverse phase separation with a 2 cm trap column (5 μm, ID 100 μm) and a 10 cm analytical column (3 μm, ID 75 μm) (EASY column, Thermo). The columns were mounted in a Thermo Scientific Nanospray Flex Ion Source with liquid junction using a steel emitter. Trap column and analytical columns were equilibrated with 100 % buffer A prior to each analysis. The eluents used were buffer A and buffer B (AcN, 5% H_2_O, 0.1% HCOOH). Peptides were eluted from the column using the following gradient: linear 0 - 40% B for 40 minutes, linear 40 - 100% for 5 minutes followed by isocratic 100% B for 45- 53minutes. For MS ionization a spray voltage of 1700 V was applied and all peptides were monitored in the positive ionization mode. Capillary temperature was set to 200° C. Selectivity for both Q1 and Q3 was 0.7 (FWHM). Argon was used as collision gas and the pressure in Q2 was set to 1.5 mTorr. The collision energy for each of the transitions was calculated using Skyline (version 2.1.0.4936). A total of 504 transitions with a cycle time of 4.0 seconds were used in the scheduled SRM method monitoring both the heavy and light peptides. MS-raw files were imported into Skyline and each file was quality checked by visual inspection of retention time and ranking of transitions for each peptide.
